# Acute Lower Respiratory Infection in Childhood and Household Fuel Use in Bhaktapur, Nepal

**DOI:** 10.1289/ehp.1205491

**Published:** 2013-03-19

**Authors:** Michael N. Bates, Ram K. Chandyo, Palle Valentiner-Branth, Amod K. Pokhrel, Maria Mathisen, Sudha Basnet, Prakash S. Shrestha, Tor A. Strand, Kirk R. Smith

**Affiliations:** 1 Division of Environmental Health Sciences, School of Public Health, University of California, Berkeley, Berkeley, California, USA; 2 Centre for International Health, University of Bergen, Bergen, Norway; 3 Department of Infectious Disease Epidemiology, Statens Serum Institut, Copenhagen, Denmark; 4 Department of Microbiology and Infection Control, University Hospital of North Norway, Tromsø, Norway; 5 Child Health Department, Institute of Medicine, Tribhuvan University, Kathmandu, Nepal; 6 Medical Microbiology, Department of Laboratory Medicine, Innlandet Hospital Trust, Lillehammer, Norway; 7 Division of Infectious Disease Control, Norwegian Institute of Public Health, Oslo, Norway

**Keywords:** biomass, case–control study, cooking, heating, household air pollution, kerosene, pneumonia

## Abstract

Background: Globally, solid fuels are used by about 3 billion people for cooking. These fuels have been associated with many health effects, including acute lower respiratory infection (ALRI) in young children. Nepal has a high prevalence of use of biomass for cooking and heating.

Objective: This case–control study was conducted among a population in the Bhaktapur municipality, Nepal, to investigate the relationship of cookfuel type to ALRI in young children.

Methods: Cases with ALRI and age-matched controls were enrolled from an open cohort of children 2–35 months old, under active monthly surveillance for ALRI. A questionnaire was used to obtain information on family characteristics, including household cooking and heating appliances and fuels. The main analysis was carried out using conditional logistic regression. Population-attributable fractions (PAF) for stove types were calculated.

Results: A total of 917 children (452 cases and 465 controls) were recruited into the study. Relative to use of electricity for cooking, ALRI was increased in association with any use of biomass stoves [odds ratio (OR) = 1.93; 95% CI: 1.24, 2.98], kerosene stoves (OR = 1.87; 95% CI: 1.24, 2.83), and gas stoves (OR = 1.62; 95% CI: 1.05, 2.50). Use of wood, kerosene, or coal heating was also associated with ALRI (OR = 1.45; 95% CI: 0.97, 2.14), compared with no heating or electricity or gas heating. PAFs for ALRI were 18.0% (95% CI: 8.1, 26.9%) and 18.7% (95% CI: 8.4%–27.8%), for biomass and kerosene stoves, respectively.

Conclusions: The study supports previous reports indicating that use of biomass as a household fuel is a risk factor for ALRI, and provides new evidence that use of kerosene for cooking may also be a risk factor for ALRI in young children.

Solid fuels, consisting of coal and biomass (crop waste, wood, and animal dung), are used by nearly 3 billion people around the world for cooking and sometimes heating [Bonjour et al., in press; UNDP (United Nations Development Program)–WHO (World Health Organization) 2009]. These fuels are often burned in stoves, without chimneys or hoods and with little or no ventilation, and are a major source of household air pollution (HAP). Use of these fuels has been associated with a wide range of health effects, particularly in women, who are most exposed because they usually do the cooking (Bruce et al. 2000; Smith et al. 2004). Also particularly highly exposed are young children who spend most of their time with their mothers. Epidemiologic studies have produced evidence that cooking with biomass is associated with acute lower respiratory infection (ALRI) or pneumonia in young children, possibly through an immune suppressant mechanism (Dherani et al. 2008; Smith et al. 2011). ALRI is the major killer of children < 5 years of age in developing countries, and has been estimated to be responsible for > 2 million deaths per year (Black et al. 2010). It has also been estimated that solid fuel use is responsible for 30% of these cases globally, and for 38% of cases in the WHO’s South-East Asian Region D, of which Nepal is a part (Niessen et al. 2009).

In Nepal, where the present study took place, many families use biomass for cooking or heating, and there can be high indoor concentrations of combustion products. We know of only one previous investigation of ALRI in relation to stove type in Nepal. This study, in the Dhading district, attributed approximately 50% of ALRI in children < 5 years of age to household use of solid fuel-burning stoves (Dhimal et al. 2010).

This case–control study was conducted in an area of Nepal with a balanced distribution of primary cookfuel types—electricity, liquified petroleum gas (LPG), kerosene, and biomass—with the primary objective of investigating the relationships of cookfuel types to ALRI and estimating population-attributable fractions.

## Methods

The study was carried out as an adjunct to a case–control study designed primarily to investigate respiratory viruses in children with and without pneumonia (Mathisen et al. 2010). Cases and controls were enrolled from an open cohort of children < 3 years old, who were under active monthly surveillance for respiratory illness in Bhaktapur, a small city (population about 72,000) 13 km east of Kathmandu, the capital of Nepal. The total population of Bhaktapur district is about 225,000, with a population density of 1,895/km^2^ (Government of Nepal 2001). Residents are farmers with fields in the surrounding area, semiskilled and unskilled laborers, and daily wage earners. There is one highway with moderate traffic on the border of the municipality and some narrow roads, mainly for motorcycles and tractors, inside. Around the municipality, there are about 10 brick kilns, which operate mainly during winter and summer and contribute to outdoor air pollution in the area.

Bhaktapur municipality represents an intermediate type between an ancient and modern city. The houses are usually joined together forming a courtyard. Most houses have traditional architecture of two or three stories and three or four rooms. The external walls are of brick with cement or mud. Roofs are usually constructed with concrete, corrugated sheet metal, or brick tiles. Floors are constructed mainly with packed mud or concrete. Of 917 study participants, 236 (26%) had primary biomass fuel stoves. Of these, 186 (79%) had a traditional open-fire mud stove, called a chulo, with 1–3 potholes. Forty-nine participants (21%) had a rice-husk mud stove without a chimney or hood.

Trained fieldworkers referred children with respiratory complaints to the study clinic at the Siddhi Memorial Hospital inside Bhaktapur municipality. Self-referrals were also accepted from the study population. Study physicians classified ALRIs according to standard WHO criteria (WHO 2005). ALRI was defined as cough or breathing difficulty combined with fast breathing (i.e., > 50 breaths/min for children 2–11 months of age or > 40 breaths/min for children ≥ 12 months of age). Severe ALRI was defined as cough or breathing difficulty accompanied by lower chest wall indrawing. Excluded from the study were cases with other severe illness, documented tuberculosis or congenital heart disease, dysentery, severe anemia (hemoglobin < 7mg/L), severe malnutrition [< 70% median weight for height or length (National Center for Health Statistics 1976)], cough for > 14 days, or having received antibiotics within the preceding 48 hr.

Potential controls, matched to cases by age in months, were randomly selected from a list of children under surveillance that was updated monthly. The surveillance was based on a baseline census conducted before the study began. Using data from the census, we generated a list of all children < 3 years of age and updated the list by identifying newborn babies and excluding children who had reached 36 months of age, moved away from the area, or left the cohort for other reasons. Fieldworkers visited homes of potential controls and requested consent from parents for their child’s participation. If consent was obtained, the child was examined for eligibility in the study clinic. Eligibility included confirmation that the child did not have ALRI and application of the same exclusion criteria as for cases. Although we measured hemoglobin concentration in all cases, we measured it only in those controls with suspected severe anemia. So it is possible that some controls with severe anemia were retained in the study.

When parents of a potential control child refused participation, another child’s family was approached. For the larger study involving viral prevalence, from which these cases and controls were drawn, 1,955 potential controls were approached, with a refusal rate of about 8% (Mathisen et al. 2010). From the case group the refusal rate was 8.5% (Mathisen et al. 2009).

During May 2006–June 2007, the household fuel use study team was notified about confirmed cases of pneumonia as they were diagnosed and about potential control households as they were identified. Subject to informed consent, a questionnaire was administered to an adult household member, usually the child’s mother, to obtain information on family characteristics, including household cooking and heating appliances, both primary and secondary. All interviews were conducted in the children’s homes, and the stoves and cookfuels used were confirmed by inspection during this visit.

Human subjects’ approvals were obtained from the institutional review boards at the University of California, Berkeley; the Institute of Medicine; Tribhuvan University Teaching Hospital; and the Regional Committee for Medical and Health Research Ethics (REK VEST), Norway.

*Statistical procedures*. Although a one-to-one age-in-months matching of cases and controls was originally sought, it was not exactly achieved, for logistical reasons and because of refusals to participate. To preserve study power, corresponding cases or controls were not eliminated from the study when a participation refusal occurred. Instead, we ran conditional logistic regression models using age in months as a matching variable, rather than individual case-to-control matchings.

We first identified all potentially appropriate covariates for which data had been collected. We selected covariates for the final model after investigating candidate variables in the following way: First we selected variables that we considered related directly to the exposure of the child (primary and secondary stove types, child in kitchen during cooking, space heating in winter, usual kitchen ventilation). Then, to identify potential confounders of the association with stove type, for each remaining variable we examined two associations: *a*) between primary stove type and potential confounder in the control group, and *b*) between the candidate variable and the outcome (ALRI) in study participants after removal of those whose families used biomass or kerosene stoves as their primary stoves. These associations were investigated without adjusting for any other variables. Any variable that predicted both primary stove type and ALRI with a *p*-value of ≤ 0.2 was included in the final model. Finally, as a precaution, we considered the model in terms of a causal diagram to ensure that we were not adjusting for anything on the causal pathway and not adjusting for a collider (Greenland et al. 1999). In addition, we evaluated the influence of adding covariates back to the selected final models on odds ratios (ORs) as a sensitivity analysis.

Some models were run after creating indicator variables for any biomass stove, any kerosene stove, and any gas stove, combining comparable primary and secondary stove types.

Population-attributable fractions (PAF) and associated confidence intervals were calculated using the aflogit command of Stata (Eide 2008). Because this procedure does not work when used with conditional logistic regression analysis, the corresponding unconditional model was used instead. The calculation of PAFs controls for covariates in the model and is based on the method described by Greenland and Drescher (1993).

## Results

A total of 917 children (452 cases and 465 controls) were recruited into the study. Table 1 shows the distribution across cases and controls of selected household characteristics, including stove/fuel types. Unadjusted ORs for ALRI and 95% CIs are also shown for each of the displayed variables. Because age was a matching factor, ORs for age are not shown.

**Table 1:** Distribution of demographic and exposure variables, with ORs for ALRI, matched for age in months, using conditional logistic regression, in Bhaktapur children, 2–36 months of age.

Variable	Controls(%)	Cases(%)	Unadjusted OR (95% CI)
Sex
Female	210 (45.2)	187 (41.4)	1.00
Male	255 (54.8)	265 (58.6)	1.16 (0.89, 1.51)
Age (months)
< 6	127 (27.3)	121 (26.8)	—
6–< 12	100 (21.5)	106 (23.5)	—
12–< 24	175 (37.6)	167 (37.0)	—
24–< 36	63 (13.6)	58 (12.8)	—
Ethnic group
Not Newari	203 (43.7)	197 (43.6)	1.00
Newari	262 (56.3)	255 (56.4)	0.99 (0.76, 1.28)
Rooms in home
1	229 (49.3)	226 (50.1)	1.00
2	41 (8.8)	60 (13.3)	1.44 (0.93, 2.24)
> 2	195 (41.9)	165 (36.6)	0.86 (0.65, 1.13)
Missing	0	1
Home ownership
Own house	231 (49.7)	214 (47.3)	1.00
Rent	234 (50.3)	238 (52.7)	1.12 (0.86, 1.46)
House sharing
Single family	245 (52.7)	260 (57.6)	1.00
Multiple families	220 (47.3)	191 (42.4)	0.83 (0.64, 1.08)
Missing	0	1	—
Domestic animals owned
No	353 (75.9)	354 (78.3)	1.00
Yes	112 (24.1)	98 (21.7)	0.87 (0.64, 1.19)
Father’s occupation
Self-employed or salary earner	183 (39.3)	155 (34.3)	1.00
Factory worker/daily wage worker	225 (48.4)	240 (53.1)	1.24 (0.94, 1.64)
Other	57 (12.3)	57 (12.6)	1.16 (0.76, 1.80)
Father’s education
More than high school	60 (12.9)	49 (10.8)	1.00
High school	228 (49.0)	199 (44.0)	1.06 (0.70, 1.63)
Primary school	163 (35.1)	184 (40.7)	1.32 (0.85, 2.04)
No school (illiterate)	14 (3.0)	20 (4.4)	1.53 (0.69, 3.36)
Mother’s work
Outside home	96 (20.7)	134 (29.7)	1.00
Housework	369 (79.3)	318 (70.3)	0.62 (0.46, 0.84)
Mother’s education
More than high school	38 (8.2)	29 (6.4)	1.00
High school	158 (34.0)	133 (29.4)	1.06 (0.62, 1.83)
Primary school	179 (38.5)	195 (43.1)	1.37 (0.81, 2.34)
No school (illiterate)	90 (19.3)	95 (21.0)	1.30 (0.74, 2.31)
Incense or mosquito coils
Not used	165 (35.5)	196 (43.4)	1.00
Used	300 (64.5)	256 (56.6)	0.73 (0.56, 0.95)
No. of smokers in household
None	188 (40.4)	175 (38.7)	1.00
1	227 (48.8)	213 (47.1)	1.03 (0.78, 1.36)
≥ 2	50 (10.8)	64 (14.2)	1.38 (0.90, 2.11)
Kitchen ceiling/roof
Metal sheet	97 (20.9)	97 (21.5)	1.00
Concrete	179 (38.5)	199 (44.0)	1.11 (0.78, 1.57)
Wood and mud	182 (39.1)	149 (33.0)	0.80 (0.56, 1.15)
Other	7 (1.5)	7 (1.5)	0.98 (0.33, 2.93)
Land ownership
No	263 (56.6)	278 (61.5)	1.00
Yes	202 (43.4)	174 (38.5)	0.79 (0.61, 1.04)
Space heating in winter
None	390 (83.9)	368 (81.4)	1.00
Electric or LPGa	18 (3.9)	9 (2.0)	0.57 (0.25, 1.29)
Wood, kerosene, or coala	57 (12.3)	75 (16.6)	1.45 (0.99, 2.11)
Daily stove use (hours)
< 2	319 (68.6)	307 (67.9)	1.00
2 to < 3	102 (21.9)	107 (23.7)	1.13 (0.83, 1.55)
≥ 3	44 (9.5)	38 (8.4)	0.90 (0.56, 1.43)
Child in kitchen during cooking
Never	117 (25.2)	82 (18.1)	1.00
Sometimes	104 (22.4)	99 (21.9)	1.35 (0.90, 2.02)
All the time	244 (52.5)	271 (60.0)	1.56 (1.12, 2.17)
Lighting when electricity fails
Candles	255 (54.8)	250 (55.3)	1.00
Emergency light	19 (4.1)	16 (3.5)	0.83 (0.42–1.66)
Kerosene wick lamp	186 (40.0)	185 (40.9)	1.02 (0.78, 1.3)
None or other	5 (1.1)	1 (0.2)	0.17 (0.02, 1.6)
Kitchen size
Large or medium	330 (71.0)	285 (63.6)	1.00
Small or very small	135 (29.0)	163 (36.4)	1.45 (1.09, 1.92)
Missing	0	4
Usual kitchen ventilation
Both doors and windows open	384 (82.6)	347 (76.8)	1.00
Either doors or windows open	77 (16.6)	102 (22.6)	1.45 (1.04, 2.03)
Neither open	4 (0.9)	3 (0.7)	0.88 (0.19, 4.02)
Primary stove fuel
Electricity	118 (25.4)	78 (17.3)	1.00
Gas	138 (29.7)	123 (27.2)	1.35 (0.92, 1.97)
Kerosene	94 (20.2)	127 (28.1)	2.13 (1.43, 3.17)
Biomass	115 (24.7)	124 (27.4)	1.69 (1.14, 2.49)
Secondary stove fuel
Electricity/none	347 (74.6)	338 (74.8)	1.00
Gas	19 (4.1)	16 (3.5)	0.86 (0.43, 1.73)
Kerosene	46 (9.9)	51 (11.3)	1.12 (0.73, 1.72)
Biomass	51 (11.0)	47 (10.4)	0.96 (0.63, 1.48)
Other	2 (0.4)	0	—
aHeating source: electric (n = 22), LPG (n = 5), wood (n = 127), kerosene (n = 3), coal (n = 2).

Several HAP exposure–related variables were associated with ALRI in the bivariate analyses. These include having a child in the kitchen when cooking, having a small kitchen, having either doors or windows (but not both) open while cooking, and using wood, kerosene, or coal for heating. Among primary stoves, kerosene burners had the highest OR relative to electric stoves, followed by biomass stoves and then gas stoves. There was no evidence of increased risk associated with secondary stoves when households using either electric secondary stoves or having no secondary stove were used as the baseline category. However, inspection of the joint distribution of primary and secondary stoves (Table 2) suggested associations between ALRI and the type of secondary stove could be negatively confounded by primary stoves: Households with no secondary stove or where the secondary stove was electric were much more likely to have primary stoves fueled by either kerosene or biomass than were other secondary stove type users. Fifty-five percent of primary electric stove-using households had biomass- or kerosene-fueled secondary stoves, compared with < 16% in all the other primary stove categories.

**Table 2:** Joint distribution of primary and secondary stove types across participating households, Bhaktapur, Nepal.

Secondary stove	Primary stove [n (%)]				
	Electricity	Gas	Kerosene	Biomass	Total
Electricity or no secondary stove	62 (31.6)	222 (85.1)	207 (93.7)	194 (81.2)	685 (74.7)
Gas	21 (10.7)	5 (1.9)	1 (0.5)	8 (3.4)	35 (3.8)
Kerosene	44 (22.5)	27 (10.3)	1 (0.5)	25 (10.5)	97 (10.6)
Biomass	69 (35.2)	6 (2.3)	11 (5.0)	12 (5.0)	98 (10.7)
Undefined	0 (0)	1 (0.4)	1 (0.5)	0 (0)	2 (0.2)
Total	196 (100)	261 (100)	221 (100)	239 (100)	917 (100)

Other than those factors, only a few of the other household characteristics in Table 1 showed evidence of association with child ALRI. Lower education of both the father and the mother and having two or more smokers in the household were associated with increased ORs. ALRI was less likely among children whose mother did housework rather than work outside the home, and was less likely if incense or mosquito coils were burned inside the home.

Included in our final model (Table 3) were the exposure-related variables: primary and secondary stove types, usual kitchen ventilation, child in kitchen during cooking, and space heating used in winter. Because they were identified as potential confounders of the primary stove–ALRI relationship, we also included mother’s education, mother’s occupation, having one or more family members who smoke indoors, and living in a single-family dwelling or sharing a house. Categorization in the model was as shown in Table 1.

**Table 3:** Exposure-related variables in final conditional multivariate logistic regression model for ALRI, in Bhaktapur children, 2–35 months of age.

Variable	ORa (95% CI)
Primary stove fuel
Electricity	1.00
Gas	1.71 (1.08, 2.72)
Kerosene	2.33 (1.40, 3.86)
Biomass	2.13 (1.34, 3.41)
Secondary stove fuel
Electricity or no secondary stove	1.00
Gas	1.52 (0.72, 3.22)
Kerosene	1.50 (0.93, 2.43)
Biomass	1.67 (0.99, 2.84)
Child in kitchen during cooking
Never	1.00
Sometimes	1.30 (0.85, 1.99)
All the time	1.60 (1.08, 2.35)
Space heating in winter
Electric, LPG, or nothing	1.00
Wood, kerosene or coal	1.45 (0.97, 2.14)
Usual kitchen ventilationb
Both doors and windows open	1.00
Either doors or windows open	1.42 (0.99, 2.05)
aModel includes the variables in the table, as well as mother’s education, mother’s occupation, family members smoke indoors, and single-family dwelling or sharing a house. bToo few participants with neither doors nor windows open for inclusion in model (Table 1).	

Adjusted odds ratios for non-electric primary and secondary stoves were higher than unadjusted estimates, particularly for secondary stoves—reflecting adjustment for confounding by primary stoves. Increased risks were associated with use of all three types of fuel-using primary cookstoves: biomass (OR = 2.13; 95% CI: 1.34, 3.41), kerosene (OR = 2.33; 95% CI: 1.40, 3.86), and gas (OR = 1.71; 95% CI: 1.08, 2.72). ORs were also positive, though not statistically significant, for the three types of secondary stoves.

Relative to either use of an electric or LPG space heater or no use of space heating (many people in Nepal just wear more clothing during cold temperatures), use of wood, coal, or kerosene for space heating in winter was associated with an OR of 1.45 (95% CI: 0.97, 2.14). Ninety-six percent (127 of 132) of those households used wood.

The adjusted OR for ALRI was positive for having either doors or windows open during cooking but not both (OR = 1.42; 95% CI: 0.99, 2.05), compared with having both windows and doors open, and the adjusted OR for children present in the kitchen all of the time during cooking, compared with never being present during cooking (OR = 1.60; 95% CI: 1.08, 2.35), was higher than the OR for being present in the kitchen some of the time (OR = 1.30; 95% CI: 0.85, 1.99).

In relation to the proportion of time the child spent in the kitchen during cooking, we found marked differences across primary stove types: For kerosene the proportion of children reported to be in the kitchen all of the time was 89%, compared with 29% for biomass (47% for electricity and 60% for gas). There was much less variation of ventilation (doors or windows open during cooking) across stove types than was observed for having the child in the kitchen. We therefore carried out analyses, similar to that of the main model, stratified by the variable for having a child in the kitchen during cooking. Table 4 shows the results. In the situation when the child is never in the kitchen, ORs are quite variable with broad confidence intervals, including the null. When the child is in the kitchen, either some of the time or all of the time during cooking, ORs are appreciably higher, with confidence intervals that exclude the null.

**Table 4:** Results of stratified analysis of the main stove type, by how often the child was in the kitchen during cooking.

Child in kitchen during cooking	n	OR (95% CI)		
		Gas	Kerosene	Biomass
Never	191	1.61 (0.44, 5.91)	0.35 (0.04, 2.66)	1.72 (0.54, 5.47)
Sometimes	189	3.62 (1.07, 12.27)	9.12 (1.35, 61.5)	2.66 (0.90, 7.86)
Always	511	1.97 (1.05, 3.72)	2.99 (1.56, 5.71)	2.17 (1.06, 4.44)

We carried out extensive sensitivity testing by adding covariates to the final model, but the ORs for primary fuel types changed relatively little whichever the additional covariates. For example, in a model with all covariates in Table 1, the ORs for primary stoves were as follows: gas (OR = 1.54; 95% CI: 0.93, 2.56), kerosene (OR = 2.14; 95% CI: 1.25, 3.67); and biomass (OR = 2.00; 95% CI: 1.22, 3.29), which are not substantially different from those in Table 3.

We also created indicator variables for any biomass stove, any kerosene stove and any gas stove, combining comparable primary and secondary stove types, and ran these in a model adjusted for the covariates in the final model (Table 3). For biomass stoves, the OR was 1.93 (95% CI: 1.24, 2.98), for kerosene stoves, 1.87 (95% CI: 1.24, 2.83), and for gas stoves 1.62 (95% CI: 1.05, 2.50).

PAFs associated with these combined stove variables were calculated after adjustment for the covariates in the final model. For ALRI in the population < 3 years of age of Bhaktapur, PAFs were as follows: biomass stoves, 18.0% (95% CI: 8.1, 26.9%), kerosene stoves, 18.7% (95% CI: 8.4, 27.8%), and gas stoves, 12.0% (95% CI: 2.5, 20.7%). For space heating with wood, kerosene, or coal, the PAF was 5.0% (95% CI: –0.6, 10.3%). The total estimated PAF for all cooking and heating fuels was 49.0% (95% CI: 25.6, 65.1%). These PAFs were calculated using the aflogit procedure following an unconditional logistic regression model that included all covariates used in the conditional model plus age. This model produced results similar to those from the conditional model. For example, for biomass and kerosene stoves, corresponding unconditional ORs were 1.97 (95% CI: 1.26, 3.07) and 1.91 (95% CI: 1.26, 2.91), respectively.

## Discussion

The database used for this analysis is possibly unique. It contains a roughly equal four-way split of households that use electricity, gas, biomass, and kerosene as their primary cookfuel. Importantly, the existence of a substantial number of houses using electric stoves provides a good baseline against which the effect of fuel-consuming stoves can be estimated. All three fuel-using stove types were positively associated with ALRI. Of particular note, the OR for kerosene primary stoves, compared with electric stoves (2.33; 95% CI: 1.40, 3.86), is comparable to or greater than that for biomass stoves (2.13; 95% CI: 1.34, 3.41).

Another unusual feature of this study is that it collected and analyzed data on secondary stoves. Most other investigations of health effects of household cookfuel in developing countries have focused on primary stoves. However, as this analysis has shown, secondary stoves may be important predictors of health risk in their own right.

Previous studies have produced evidence that biomass-burning stoves are associated with increased relative risk estimates for ALRI. A meta-analysis of 24 studies produced a summary estimate of 1.78 (95% CI: 1.45, 2.18) for the relationship between household use of solid fuels (wood, dung, charcoal, and coal), relative to use of fuels considered “clean” (electricity, gas, or kerosene), and ALRI in children < 5 years of age (Dherani et al. 2008). More recent studies have found ALRI risks of a similar magnitude associated with solid fuel use (Bautista et al. 2009; Rehfuess et al. 2009). This compares with our OR of 2.13 (Table 3) for having a main stove that used biomass. The difference may reflect that our baseline category is electricity, whereas most of the studies included in the meta-analysis used gas or liquid fuels as their baseline.

To our knowledge, only one previous investigation has examined kerosene-burning stoves as a possible risk factor for ALRI. In a study of ALRI in 642 children < 1 year of age in two slums in Delhi, India, the OR for kerosene use in one slum was 1.98 (95% CI: 1.14, 3.45), but in the other slum was 0.95 (95% CI: 0.59, 1.54) (Sharma et al. 1998). The authors provided no explanation for the difference in results. There have been few epidemiologic studies that have investigated kerosene stove use as a possible risk factor for any respiratory disease. However, a recent study of risk factors for pulmonary tuberculosis in Nepal reported a substantially stronger association with kerosene cookstoves (OR = 3.4; 95% CI: 1.0, 11), than with biomass-burning cookstoves (OR = 1.2; 95% CI: 0.5, 3.1) (Pokhrel et al. 2010). In the same study, the OR for use of simple kerosene lamps, relative to electric lighting, was 9.43 (95% CI: 1.45, 61).

We also note the elevated ORs for cooking with gas. Because most studies investigating associations between cooking with biomass and ALRI have been carried out using LPG in the reference fuel category, there are few, if any, available comparisons. In some countries, however, LPG use has been associated with increased respiratory risk (Moshammer et al. 2010), suggesting that its use may not be entirely benign. Other studies using cooking with electricity as a baseline category are needed to ascertain whether our finding for gas cooking represents a real risk increase.

Although the responsible components of cooksmoke that induce respiratory problems are unknown, it has been well established by a number of studies that the amount of indoor air pollution from particulate matter emitted by kerosene stoves is appreciably less than that associated with biomass-burning stoves (Saksena et al. 2003). In one study, however, kerosene stove– and wood stove–using cooks had approximately the same levels of personal exposures to particulate matter, despite kitchen PM concentrations for wood users being approximately twice that of kerosene users (Saksena et al. 2003). The authors suggested that this difference occurred because kerosene users cook for longer periods than wood users, are more likely to cook indoors, and spend more time in closer proximity to the stove. Because young children are likely to spend most of their waking time close to their mothers, our findings on the relative proportions of time children spend in the kitchen in families with primary biomass or kerosene stoves are consistent with Saksena’s observations.

Alternative explanations for our results, particularly those for kerosene, need to be considered. Information bias seems unlikely because the ALRI was clinically confirmed and interviews were carried out in homes, permitting interviewers to confirm the accuracy of stove reporting. Use of primary and secondary stove/fuel types as categorical variables is a somewhat crude representation of exposures within a home, because it does not account for relative frequency of use or for other fuel-using devices, such as lamps or additional stoves. This would lead to exposure misclassification, which, assuming it to be nondifferential, would most likely bias results toward the null. Thus, it would not provide a likely explanation for our findings.

In general, the type of cookfuel used by a household is related to socioeconomic status, which is commonly associated with ALRI. It is therefore possible that there may be residual confounding by unmeasured factors related to socioeconomic status. Our analysis took into account a range of socioeconomic factors, including parental occupation and education, land ownership, house size and construction materials, and house ownership.

Selection bias arising from study participation must be considered because not all families invited agreed to participate. For this to be a factor, the ratio of willingness to participate by kerosene cookstove–using case families to willingness to participate by kerosene cookstove–using control families would need to have been greater than the corresponding ratio for electric cookstove–using case and control families. We have no data that would allow us to judge whether this could have been the case. Because there was a low refusal rate of both case and control families (8–9%), however, this seems unlikely to have been a significant source of bias. Notably, we excluded cases of ALRI who had received antibiotics within the 48 hr before assessment, which would have resulted in exclusion of more severe cases. The consequence for the described associations between fuel type and ALRI is unclear.

For the PAF calculation we have assumed that the proportion of controls exposed would estimate the proportion exposed in the population < 3 years of age. The baseline census and the subsequent monthly active surveillance in all the neighborhoods of Bhaktapur municipality, including the free treatment offered by our study clinic, made the project well known in the area and encouraged families to use our services. Due to delay in registration after birth, we may have failed to include in our surveillance some of the youngest children, as well as some children from migrant families, who come to the area in search of work and have limited social networks in the local community. These groups would, however, comprise a small proportion of the population < 3 years old, and any bias would be nondifferential with regard to case and control status.

## Conclusions

Our investigation supports other findings that use of biomass as a household fuel is a risk factor for ALRI, and provides new evidence that the types of kerosene and kerosene stoves used in Nepal for cooking may also be a risk factor for ALRI in young children. It also suggests that keeping children out of the kitchen during cooking is useful in reducing risk. These results add to the small body of evidence that kerosene, sometimes viewed as a “modern” or “clean” fuel, may be much less benign than previously assumed. The results of this study concerning possible health impacts of kerosene need to be confirmed in other settings. More generally, it is important that investigators studying health effects of household fuel use consider kerosene as a distinct fuel category, deserving specific investigation, rather than combining it with other fuels in the reference “clean” fuel category, as has often been done in the past. If the health implications of household kerosene use are confirmed, it would call into question the common practice in developing countries of providing kerosene subsidies to poor households.

## References

[r1] Bautista LE, Correa A, Baumgartner J, Breysse P, Matanoski GM (2009). Indoor charcoal smoke and acute respiratory infections in young children in the Dominican Republic.. Am J Epidemiol.

[r2] Black RE, Cousens S, Johnson HL, Lawn JE, Rudan I, Bassani DG (2010). Global, regional and national causes of child mortality in 2008: a systematic analysis.. Lancet.

[r3] Bonjour S, Adair-Rohani H, Wolf J, Bruce N, Mehta S, Pruss-Ustan A Solid fuel use for household cooking: country and regional estimates for 1980–2010.. Environ Health Perspect..

[r4] Bruce N, Perez-Padilla R, Albalak R (2000). Indoor air pollution in developing countries: a major environmental and public health challenge.. Bull WHO.

[r5] Dherani M, Pope D, Mascarenhas M, Smith KR, Weber M, Bruce N (2008). Indoor air pollution from unprocessed solid fuel use and pneumonia risk in children aged under five years: a systematic review and meta-analysis.. Bull WHO.

[r6] Dhimal M, Dhakal P, Shrestha N, Baral K, Maskey M (2010). Environmental burden of acute respiratory infection and pneumonia due to indoor smoke in Dhading.. J Nepal Health Res Counc.

[r7] Eide GE (2008). Attributable fractions for partitioning risk and evaluating disease prevention: a practical guide.. Clin Respir J.

[r8] Government of Nepal. (2001). National Census of Nepal. Central Bureau of Statistics.

[r9] Greenland S, Drescher K (1993). Maximum likelihood estimation of the attributable fraction from logistic models.. Biometrics.

[r10] Greenland S, Pearl J, Robins JM (1999). Causal diagrams for epidemiologic research.. Epidemiology.

[r11] Mathisen M Strand TA Sharma BN Chandyo RK Valentiner-Branth P Basnet S 2009 RNA viruses in community-acquired childhood pneumonia in semi-urban Nepal: a cross-sectional study. BMC Med 7 35; 10.1186/1741-7015-7-35 [Online 27 July 2009] 19635124 PMC2727531

[r12] Mathisen M, Strand TA, Valentiner-Branth P, Chandyo RK, Basnet S, Sharma BN (2010). Respiratory viruses in nepalese children with and without pneumonia: a case-control study.. Pediatr Infect Dis J.

[r13] Moshammer H, Fletcher T, Heinrich J, Hoek G, Hruba F, Pattenden S (2010). Gas cooking is associated with small reductions in lung function in children.. Eur Respir J.

[r14] National Center for Health Statistics. (1976). NCHS growth charts, 1976.. Mon Vital Stat Rep.

[r15] Niessen L, ten Hove A, Hilderink H, Weber M, Mulholland K, Ezzati M (2009). Comparative impact assessment of child pneumonia interventions.. Bull WHO.

[r16] Pokhrel AK, Bates MN, Verma SC, Joshi HS, Sreeramareddy CT, Smith KR (2010). Tuberculosis and indoor biomass and kerosene use in Nepal: a case–control study.. Environ Health Perspect.

[r17] Rehfuess EA, Tzala L, Best N, Briggs DJ, Joffe M (2009). Solid fuel use and cooking practices as a major risk factor for ALRI mortality among African children.. J Epidemiol Community Health.

[r18] Saksena S, Singh P, Prasad RK, Prasad R, Malhotra P, Joshi V (2003). Exposure of infants to outdoor and indoor air pollution in low-income urban areas: a case study of Delhi.. J Expo Anal Environ Epidemiol.

[r19] Sharma S, Sethi GR, Rohtagi A, Chaudhary A, Shankar R, Bapna JS (1998). Indoor air quality and acute lower respiratory infection in Indian urban slums.. Environ Health Perspect.

[r20] Smith KR, McCracken JP, Weber MW, Hubbard A, Jenny A, Thompson LM (2011). Effect of reduction in household air pollution on childhood pneumonia in Guatemala (RESPIRE): a randomised controlled trial.. Lancet.

[r21] Smith KR, Mehta S, Maeusezahl-Feuz M. (2004). Indoor smoke from household solid fuels.

[r22] UNDP (United Nations Development Programme)–WHO (World Health Organization). (2009). The Energy Access Situation in Developing Countries: A Review Focusing on the Least Developed Countries and sub-Saharan Africa. New York:United Nations Development Program.

[r23] WHO (World Health Organization). (2005). Model IMCI Handbook: Integrated Management of Childhood Illness.

